# Comparative study of the gut microbial communities collected by scraping and swabbing in a fish model: a comprehensive guide to promote non-lethal procedures for gut microbial studies

**DOI:** 10.3389/fvets.2024.1374803

**Published:** 2024-03-22

**Authors:** Alberto Ruiz, Silvia Torrecillas, Elena Kashinskaya, Karl B. Andree, Mikhail Solovyev, Enric Gisbert

**Affiliations:** ^1^Aquaculture Program, Centre de La Ràpita, Institut de Recerca i Tecnologia Agroalimentàries (IRTA), La Ràpita, Spain; ^2^Institute of Systematics and Ecology of Animals, Siberian Branch of the Russian Academy of Sciences, Novosibirsk, Russia; ^3^A.N. Severtsov Institute of Ecology and Evolution, Russian Academy of Sciences, Moscow, Russia; ^4^Biological Institute, Tomsk State University, Tomsk, Russia

**Keywords:** fish microbiota, mucosa, intestine, sampling procedure, animal welfare, reduction, refinement, *Mycoplasma*

## Abstract

In the present study, we propose the use of swabs in non-lethal sampling procedures to collect the mucosa-adhered gut microbiota from the posterior intestine of fish, and therefore, we compare the bacterial communities collected by conventional scraping and by swabbing methods. For this purpose, samples of the posterior intestine of rainbow trout (*Oncorhynchus mykiss*) were collected first using the swabbing approach, and after fish euthanasia, by mucosa scraping. Finally, bacterial communities were compared by 16S rRNA gene Illumina sequencing. Results from the current study revealed that similar values of bacterial richness and diversity were found for both sampling procedures. Similarly, there were no differences between procedures when using qualitative metrics (Jaccard and unweighted UniFrac) for estimating inter-individual diversity, but the quantitative metrics (Bray-Curtis and weighted UniFrac) showed a higher dispersion when samples were obtained by swabbing compared to scraping. In terms of bacterial composition, there were differences in abundance for the phyla Firmicutes and Proteobacteria. The cause of these differential abundances may be the inability of the swab to access to certain areas, such as the basal region of the intestinal villi. Moreover, swabbing allowed a higher representation of low abundant taxa, which may also have an important role in host microbiome regardless of their low abundance. Overall, our results demonstrate that the sampling method is a factor to be considered in experimental design when studying gut bacterial communities to avoid potential biases in the interpretation or comparison of results from different studies. In addition, the advantages and disadvantages of each procedure (swabbing *vs* scraping) are discussed in detail, concluding that swabbing can be implemented as a reliable and non-lethal procedure for posterior gut microbiota studies, which is of particular interest for animal welfare and the 3Rs principle, and may offer a wide range of novel applications.

## 1 Introduction

The fish gut microbiota has developed intimate relationships with the host, especially the autochthonous microbiota that colonizes the intestinal mucosa, being involved in a wide range of functions, such as feed digestion, nutrient metabolism, energy homeostasis, barrier function, immune system modulation, neural development, or regulation of the endocrine system, among others (1). Such involvement in health has made the fish gut microbiome an increasingly frequent target of many research studies during the last decades (2). The fast-growing interest in the gut microbiota has made possible to discern the specific functionality of certain bacteria regarding their host, unraveling some bacterial taxa as possible markers of fish health and condition (3, 4). In this sense, the manipulation of the fish microbiota has provided the aquaculture industry with many opportunities to improve production (5), such as the use of probiotics with growth-promoting effects and/or that improve feed efficiency (6), and the prevention and control of diseases in culture systems (7). Similarly, the study of intestinal microbiota in wild fish has also many applications, as it can be used for ecological monitoring of the ecosystem health (8, 9), to understand the environmental impact of threats as climate change and human pollution (10), or to promote biodiversity conservation (11), among other applications.

Standardized approaches for sampling and data analysis are needed to ensure reproducibility and comparability of microbial results across studies (12). There is no defined consensus within the scientific community regarding the procedures to be followed when analyzing the fish gut microbiota. In this sense, Pond et al. (13) showed that a large proportion of bacteria from the intestine of rainbow trout (*Oncorhynchus mykiss*) were non-culturable and could not be identified using conventional microbiological techniques, and consequently, the vast majority of research groups have now adopted the perspective of molecular analyses (14). Nonetheless, there are other methodological parameters that are usually not considered when comparing outcomes from different studies and that may influence the results regarding the selected approach. For instance, it has been reported that fish gut microbiome results are conditioned by the storage method (15, 16), the DNA extraction protocol (16–18), the PCR conditions (19), the specific primers used for amplification and sequencing (19, 20), and the sequencing platform used (20). Regarding sample collection, there are some basic overriding factors to consider, such as the fish nutritional condition (postprandial or fasted) (21), the specific intestinal region sampled (22, 23), and the sample size (24). However, to date, there is very limited information on the influence of the sampling methodology on the results obtained for fish gut microbiota.

Two different sampling procedures are normally used to collect the microbiota associated to mucosal tissues in fish: scraping and swabbing. The scraping technique involves gently but persistently passing a spatula or scalpel against the mucosal surface to scratch and collect the mucosal layer (25–27), while swabbing consists of vigorously swiping a sterile swab in the area to be sampled to absorb the microbial content (28–30). In fish studies, both techniques have generally been used with prior sacrifice of the animal for gut dissection. However, if the size of the fish is adequate to allow the annal entry of flexible applicators with low thickness, swabbing may be implemented as a quick non-lethal sampling technique in future research studies of the fish gut microbial communities, as is done in higher vertebrates (31–33). This is of special relevance in the era of animal welfare and the 3Rs (replacement, reduction and refinement) principles in humane animal research, especially under the scopes of reduction and refinement. Thus, the objective of this work was to compare the effect of traditional scraping method versus the non-lethal swabbing technique to study the diversity, structure and composition of the microbial communities associated to the mucosa of the fish posterior intestine. For this purpose, we selected rainbow trout as a model, a salmonid freshwater fish among the most farmed species worldwide (34).

## 2 Materials and methods

### 2.1 Ethics statement

All procedures related to animal care, manipulation and sampling were carried out by trained competent personnel according to the Spanish (law 32/2007 and Royal Decree 1201/2015) and European legislation (EU2010/63) and were approved by the Generalitat of Catalunya (CEEA 219/2020) and by the Ethical Committee of the Institute of Agrifood Research and Technology (Spain).

### 2.2 Sampling

In this trial, rainbow trout with a body weight of 390.5 ± 36.8 g (mean ± standard deviation) and a standard length of 28.5 ± 0.8 cm were used. Since our purpose was to establish a microbial comparison regarding different methodological approaches, captivity fish were used, and reared under the following culture-controlled conditions, to avoid environmental factors which may have an impact on the fish microbiome (35). Fish were reared at IRTA la Ràpita (Spain) for 3 months under controlled water temperature, pH, and dissolved oxygen of 15.9 ± 0.7°C, 7.9 ± 0.2, and 8.8 ± 1.0 mg/L, in 450 L-tanks connected to an IRTAmar™ water recirculation system. Since their arrival at IRTA, rainbow trout were fed with an extruded diet (43% crude protein, 15% crude fat, 20.6 MJ/kg of energy). Two days before the sampling, fish were fasted to ensure the removal of feces from the intestinal tract in order to sample only the autochthonous mucosa-associated microbiota (21). For sampling purposes, 15 individuals from the same tank were hand-netted and anesthetized by immersion in 100 mg/L of buffered tricaine methanesulfonate (MS-222, Sigma-Aldrich, Spain). Then, the external surfaces of each fish were rinsed with absolute ethanol (100%) to avoid any potential external contamination (12). First, a sterile flocked nylon swab (MFS-96000BQ, Meidike Gene, China) was inserted anally into the fish for *ca.* 5–6 cm, noticing a first pressure when passing through the ileocecal valve, and continuing until another pressure was felt, corresponding to the wall of the intestine folding up. The swab was gently rotated in a clockwise and anticlockwise circular motion, carefully withdrawn, and the tip was placed in a sterile tube. The swab model used is specifically designed to collect large numbers of cells for clinical or research purposes, and it was selected because of its thin tip (Ø = 2.5 mm), flexible polystyrene body, breakpoint that prevents cross-contamination from other materials to cut the tip, and because of the demonstrated higher yield of flocked nylon swabs for collecting microorganisms in comparison to other types of swabs and applicators (36). After that, each fish was euthanized in a bucket with an overdose of anesthetic buffered tricaine methanesulfonate (350 mg/L of MS-222) for posterior scraping. Briefly, each fish was dissected and a section of *ca.* 5–6 cm of the posterior intestine, from the anus backward, was extracted and aseptically opened lengthwise. The inner walls were gently but insistently scraped, with a round-edge spatula, to recover the mucosal content. Both kinds of samples from each specimen were individually stored at −80°C until further DNA extraction. To avoid inter-individual variability, both types of samples (swabbed and scraped) were collected from the same specimen, as traditionally done in comparative methodological studies in higher vertebrates (37–39), ensuring that there was still enough mucus when dissecting the intestine to obtain a representative microbial sampling.

To ensure that the flocked nylon swabs would fit into the fish posterior intestine, 2 weeks before the trial, five rainbow trout from another tank were anesthetized with MS-222 (150 mg/L) and anally swabbed as described above; then, the fish were returned to their original tank. The length of the anal insertion of the swab (5–6 cm) was used as a reference for sampling the same region by scraping. No abnormal behavior, stress, or sign of disease were observed by the trained personnel of the facilities during the following days.

### 2.3 Extraction of DNA and amplicon sequencing

Extractions of DNA were performed with the DNeasy PowerSoil Pro Kit (ref. 47,016, QIAGEN, Germany) following the manufacturer instructions. In the case of the swab samples, the whole flocked nylon tips were included in the tubes until the bead-beating step (included) to ensure cell lysis and sample homogenization, whereas for the scraped samples, a total content of *ca.* 100 mg per tube were used for each DNA extraction. The concentration and purity of extracted DNA were measured in a Nanodrop-2000^®^ spectrophotometer (Thermo Fisher Scientific, United States). The DNA concentrations ranged from 80 to 200 ng/μL and the A_260_/A_280_ absorbance ratios were higher than 1.80.

The bacterial universal primers 341F (5′-CCTACGGGNGGCWGCAG-3′) and 805R (5′-GACTACHVGGGTATCTAATCC-3′) were used to amplify the V3-V4 region of the 16S rRNA gene by means of a Q5^®^ High-Fidelity DNA Polymerase (ref. M0491L, New England BioLabs, United States). A first PCR was run as follows: an initial step of 30 s at 98°C for polymerase activation and DNA denaturation, followed by 25 cycles of 10 s at 98°C, 30 s at 55°C, 30 s at 72°C, and a final extension step of 2 min at 72°C. Then, a second 8-cycle amplification was carried out in which specific barcodes were added to each template. The amplified regions were prepared for sequencing on an Illumina MiSeq Platform (2 × 300 bp paired-end) following the instructions of the 16S Metagenomic Sequencing Library Preparation guide (40). Two “mock” samples with a known bacterial composition were amplified and sequenced as positive controls, and no-template PCRs were also included and sequenced as negative controls. Raw sequencing data are available in the Sequence Read Archive (SRA) of NCBI under Bioproject accession number PRJNA1064675.

### 2.4 Data analyses

Firstly, *Cutadapt* was applied to remove forward and reverse primers from the *fastq* files using QIIME2 (v2022.2) (41). Data analyses were carried out in RStudio (v2023.06.1) (42), with the open-source package DADA2 (v1.24.0), which models and corrects errors derived from Illumina sequencing (43). The DADA2 package resolves differences at the single-nucleotide level and the end products are amplicon sequence variant (ASVs). In brief, forward and reverse reads were subjected to quality filtering and an individual and average quality threshold of 26 was established, with truncation lengths of 280 nt and 220 nt for the forward and reverse reads, respectively. Reads with an expected error higher than 2 were removed from analysis. Then, paired-end reads were merged and the sequences with an overlap length < 12 nucleotides, more than 0 mismatches, or identified as chimeras were also excluded. Resultant ASVs were taxonomically classified according to SILVA database (v138.1) (44). A bootstrapping confidence of 80% was established as a reliable cut-off (45), and the ASVs with a lower assignment percentage were classified as unassigned. Those ASVs classified as mitochondria and chloroplasts were not included in the analysis. According to the rarefaction curves obtained with the R package vegan (v2.6–4) (46), samples were rarefied to the number of reads of the sample with the lowest depth (214,663 reads per sample), which was a representative size for the ASVs present in the samples (Supplementary Figure S1) and normalized by total sum scaling (47). Rarefaction was performed in the R package microeco (v0.20.0) using the *trans_rarefy* function (48). All Good’s coverage values were ≥ 99.6% and none of the samples were discarded during the analyses.

### 2.5 Comparison of gut communities and statistical analyses

Regarding alpha diversity indices, the richness estimators of Chao1, ACE, and diversity indices of Shannon, Simpson, and Faith’s phylogenetic were calculated (49, 50) with the function *trans_alpha* of the R package microeco (48). Chao1 and ACE indices estimate ASV richness based on the number of observed ASVs and on the number of estimated missing species. For reckoning the number of missing species, the Chao1 index considers singletons and doubletons (species with a total of one or two observations, respectively), while the ACE index only takes into account the species with less than 10 observations (50). Shannon and Simpson indices estimate both species richness and evenness (degree of homogeneity in the distribution of species abundances); the Shannon index gives more weight to species richness, while the Simpson index gives more weight to species evenness (50). Faith’s phylogenetic index also estimates diversity, based on species richness, abundance, and phylogeny (49). For beta diversity, the dissimilarities among samples were estimated according to the qualitative Jaccard and unweighted UniFrac distances, and the quantitative Bray-Curtis and weighted UniFrac distances (51–53), which were represented with principal coordinate analysis (PCoA), using the function *trans_beta* of the R package microeco (48). In brief, Jaccard only considers the number of common and different ASVs among samples (51), whereas unweighted UniFrac also contemplates the phylogenetic relation between those ASVs (53). Bray-Curtis considers the frequency of each ASV, and the number of common and different ASVs (52), and weighted UniFrac considers the above plus the phylogenetic relationships among ASVs (53). Significant differences in alpha and beta diversity values (*p* ≤ 0.05) were estimated using linear mixed-effects models, with the function *lmer* of the R package lme4 (54), establishing the sampling procedure as a fixed effect, and the fish identification as a random effect, to account for repeated measures of the fish. Regarding significant differences in composition, a negative binomial generalized linear mixed-effects model was performed to minimize overdispersion and zero-inflation by means of the R package MaAsLin2 (55), establishing the sampling procedure as a fixed effect, and the fish identification as a random effect. *p*-values were corrected for multiple comparisons using Benjamini-Hochberg (BH) correction (*p* ≤ 0.05) (56).

## 3 Results and discussion

To date, very few comparative studies focused on mucosal-associated microbiota when using different sampling methods have been performed in fish mucosal tissues, such as gills (57–59), skin (60), and gut (61), which have obtained some controversial results. In the gastrointestinal tract, there has been shown evidence of a divergent composition between the bacteria weakly adhering to the intestinal mucosa collected by washing and those obtained when scraping the mucosa in European whitefish (*Coregonus lavaretus*) (61). Nonetheless, to the best of our knowledge, there are no reports yet specifically focused on comparing the microbial diversity, structure, and composition of the microbial communities from the gut-associated mucosa collected by different sampling procedures and involving the non-sacrifice of the fish. Therefore, the results presented below describe the similarities and differences found in the characterization of the fish bacterial communities from the posterior intestine by 16S rRNA gene sequencing when applying two different sampling methodologies, and we propose a non-lethal collection procedure to be used when the objectives and needs of the study allow it.

**Figure 1:**
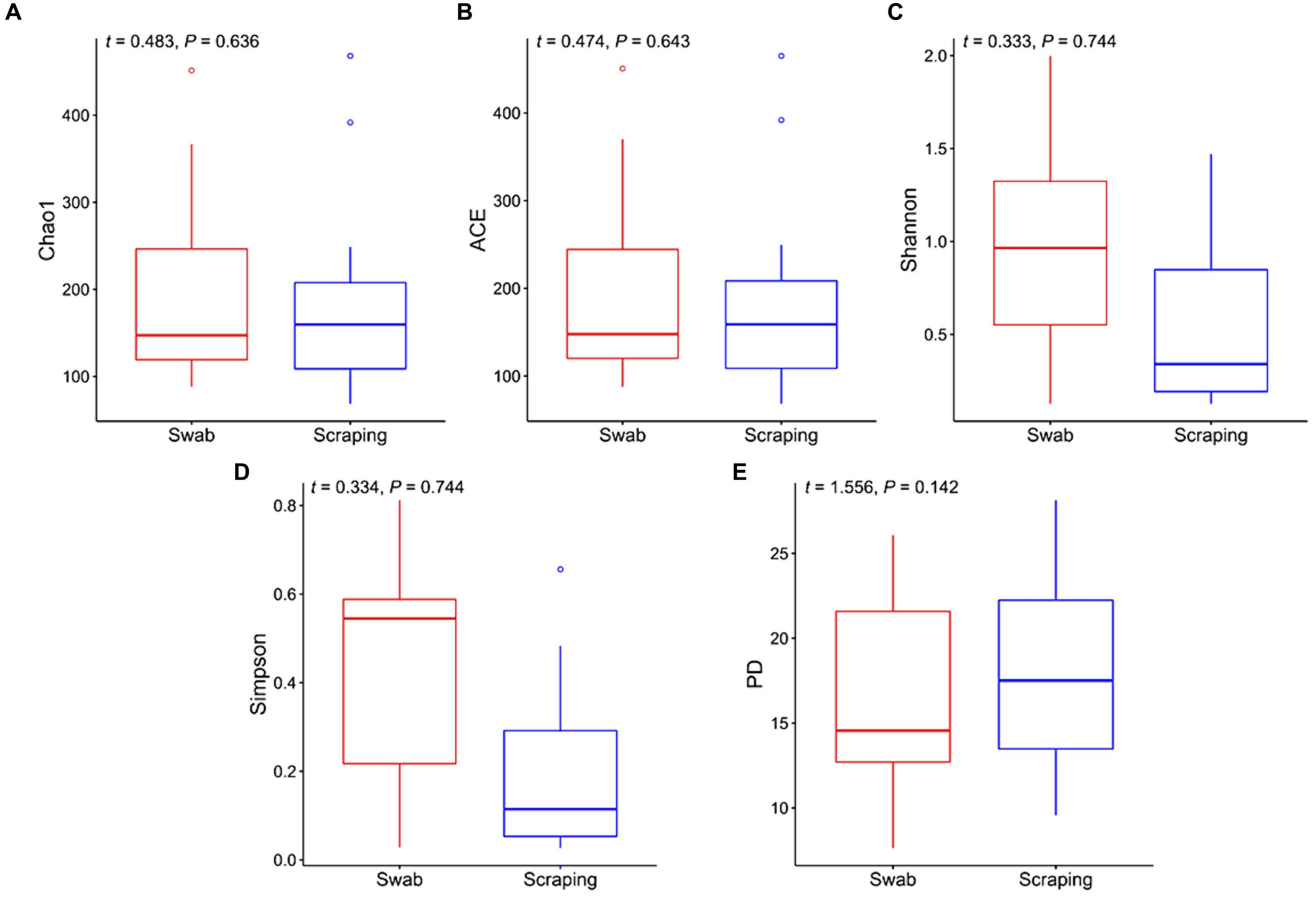
Alpha diversity indices for the bacterial communities associated to the mucosa from the posterior intestine in rainbow trout (*Oncorhynchus mykiss*) collected by swabbing and scraping (*n* = 15 per sampling method): **(A)** Chao1 index, **(B)** ACE index, **(C)** Shannon index, **(D)** Simpson index, **(E)** Faith’s phylogenetic diversity index (PD).

In the current study, we firstly compared the diversity of the bacterial communities from the posterior gut mucosa collected by swabbing and scraping using the following alpha diversity indices (Figure 1). Interestingly, a non-significant upward trend in the values of Chao1, ACE, Shannon, and Simpson was observed when the intestinal mucosa was sampled by swabbing rather than scraping (*p* > 0.05; Figures 1A–D). Similarly, Clinton et al. (57) reported higher values for Richness, Shannon and Simpson indices when sampling the Atlantic salmon (*Salmo salar*) gills by swabbing rather than by tissue excision. The former authors hypothesized that the so-called “cryptic” locations, which are zones not reached by swabbing, may have lower microbial diversity than surface mucus communities. This hypothesis may be extrapolated also to the posterior intestine, attending to the results of the present study. In addition, the phylogeny of the species found in both types of samples seemed to be similar according to the even values of Faith’s phylogenetic index between both sampling procedures (*t* = 1.56, *p* = 0.14; Figure 1E).

As mentioned above, there are not yet comparative studies between both techniques herein employed in fish, and those in higher vertebrates are scarce and mainly focused on comparing the fecal microbiota, which is transient, and the microbiota from the whole tissue or from the swabbed content, which is considered to be mainly autochthonous in fasted animals. A pair of studies in amphibians and reptiles comparing the tissue and swabbed microbiota are partly in line with the present work. Indeed, higher, but not significantly different Shannon values were observed in adult cane toads (*Rhinella marina*) when using a cloacal swab in comparison to the microbiota collected from the large intestine by squeezing (38). In striped plateau lizards (*Sceloporus virgatus*), a similar upward trend in the Shannon index was observed when using cloacal swabs rather than collecting the cloacal tissue, results that were coupled with higher richness and Faith’s phylogenetic diversity (39). In poultry, where the use of cloacal swabs as a non-invasive method of microbial sampling is more common, controversial results have been found depending on the study. Some reports in juvenile ostriches (*Struthio camelus*) and broiler chickens have reported a higher diversity in the colon and/or cecal content when sampling the specific tissue content than when using cloacal swabs (37, 62, 63). On the other hand, other works in broiler chickens and zebra finches (*Taeniopygia guttata*), have reported a very similar diversity when both methods were applied, tissue dissection of the large intestine and cloacal swabbing (64, 65). The different results among studies are not only explained by the different animal species targeted, but also by the different sampling techniques used to analyze the microbiota associated to the tissue. For instance, some of these works collected only the luminal content by squeezing the intestine (38, 63), while in other trials the specific part of the tissue was dissected for DNA extraction (39). In addition, none of the above-mentioned studies specified whether the animals were fasted prior to sampling or if the collected tissue was cleaned in order to remove the transient microbiota, so probably the majority of the sampled bacteria were allochthonous (21). This makes it more difficult to compare such results with ours, and points to the need for standardization of procedures among scientists and for comparative trials to study the effect of different procedures on mucosa-attached (autochthonous) microbiota, which is involved in long-lasting host-microbiome interactions and has a direct effect on the host physiology (66).

**Figure 2:**
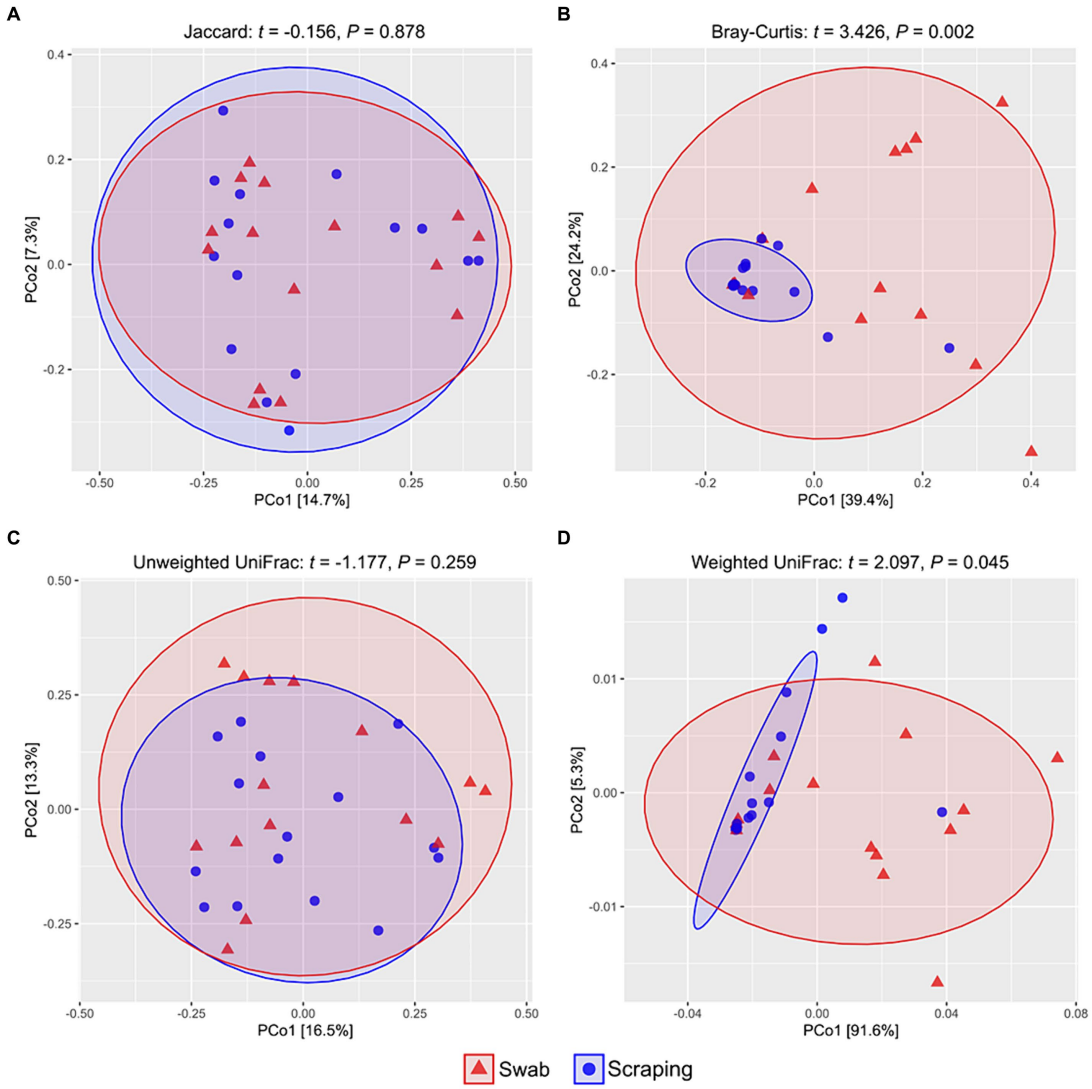
PCoA analyses showing the spatial distribution of the bacterial communities among samples from the posterior intestine mucosa of rainbow trout (*Oncorhynchus mykiss*) collected by swabbing and scraping (*n* = 15 per sampling method), based on different beta diversity metrics: **(A)** Jaccard distances, **(B)** Bray-Curtis distances, **(C)** unweighted UniFrac distances, **(D)** weighted UniFrac distances.

Regarding beta diversity metrics, there were no differences among samples obtained by swabbing and scraping when using the Jaccard distances (*t* = −0.16, *p* = 0.88; Figure 2A) and the unweighted UniFrac distances (*t* = −1.18, *p* = 0.26; Figure 2C), while a significant divergence was observed between both groups for Bray-Curtis (*t* = 3.43, *p* < 0.002; Figure 2B) and weighted UniFrac distances (*t* = 2.10, *p* = 0.045; Figure 2D). Consequently, our findings indicated a similar number of ASVs and a similar phylogenetic diversity of these ASVs when using both sampling methods, but a very different representation of the abundances of these sequences. Such results, together with the observed upward trend in the Chao1, ACE, Shannon and Simpson indices in the swabbed samples, pointed to a higher dominance of certain species on the scraped samples, while a higher diversity on the swabbed ones. Furthermore, it was notable the high dispersion observed in the swabbed samples compared to the scraped ones, which has also been reported in previous works from ostriches (37) and broiler chickens (63), as well as in human trials (67), with respect to samples obtained from the dissected tissue, cecal content and rectal mucosa, respectively. When interpreting the results of the present study, it is important to note that the higher dispersion observed when using the flocked swabs is in relation to samples obtained from collecting all the posterior gut mucosa, and in this sense, low biomass-samples usually have lower repeatability than those with higher biomass (scraping method), due to the lower concentration of bacterial DNA (68). However, a differential clustering of the bacterial communities based on Bray-Curtis distances has been observed in sockeye salmons (*Oncorhynchus nerka*) regarding their population location when collecting the microbiota of the posterior intestine by anal swab insertion (*n* = 20 per group) (69), even though such individuals were euthanized just before sampling. This means that, even though in relation to other sampling procedures the inter-individual variability of swabbed samples can be high, this method also allows to discern significant differences in beta diversity among experimental groups. In addition, knowing that a higher dispersion can be expected when using the swabbing technique rather than mucosal scraping, the sampling of a higher number of fish per experimental group than usual is recommended to reduce inter-individual variability if the purpose is to compare diversity (24), especially considering that the size of the sample population (*n*) is not such a limiting factor when using non-lethal methods.

**Figure 3:**
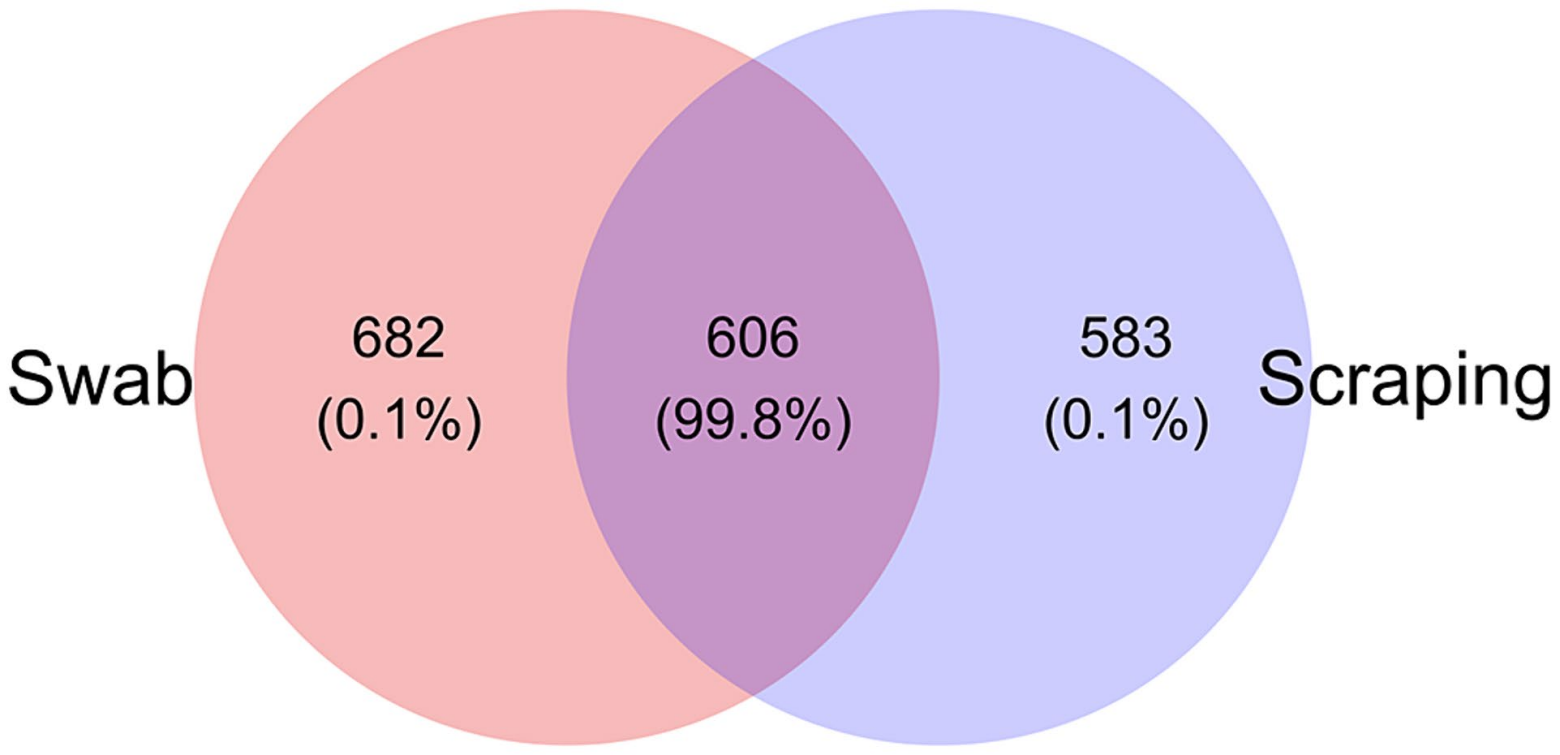
Venn diagram plotting the number of unique and shared ASVs (and relative abundance %) collected by swabbing and scraping the mucosa from the posterior intestine of rainbow trout (*Oncorhynchus mykiss*; *n* = 15 per sampling method).

Regarding microbial composition, a total of 1,288 ASVs were found among the swabbed samples, while 1,189 ASVs were observed in the scraped ones. Despite only one third of the ASVs (606 out of 1,871) were shared between the samples obtained with both procedures, it represented a total of 99.8% relative abundance (Figure 3). Meanwhile, 682 ASVs were exclusively found in the swabbed templates, with a total relative abundance of 0.1%, and 583 ASVs were unique from the scraped mucosa, comprising 0.1% relative abundance. Consequently, the divergences related to differential abundances among procedures reflected in the Bray-Curtis and weighted UniFrac distances were probably mainly a consequence of the differential abundances among shared ASVs. The similar number of exclusive ASVs identified in both groups also explained the absence of differences in the values of Chao1 and ACE indices, and when using Jaccard and unweighted UniFrac distances.

**Figure 4:**
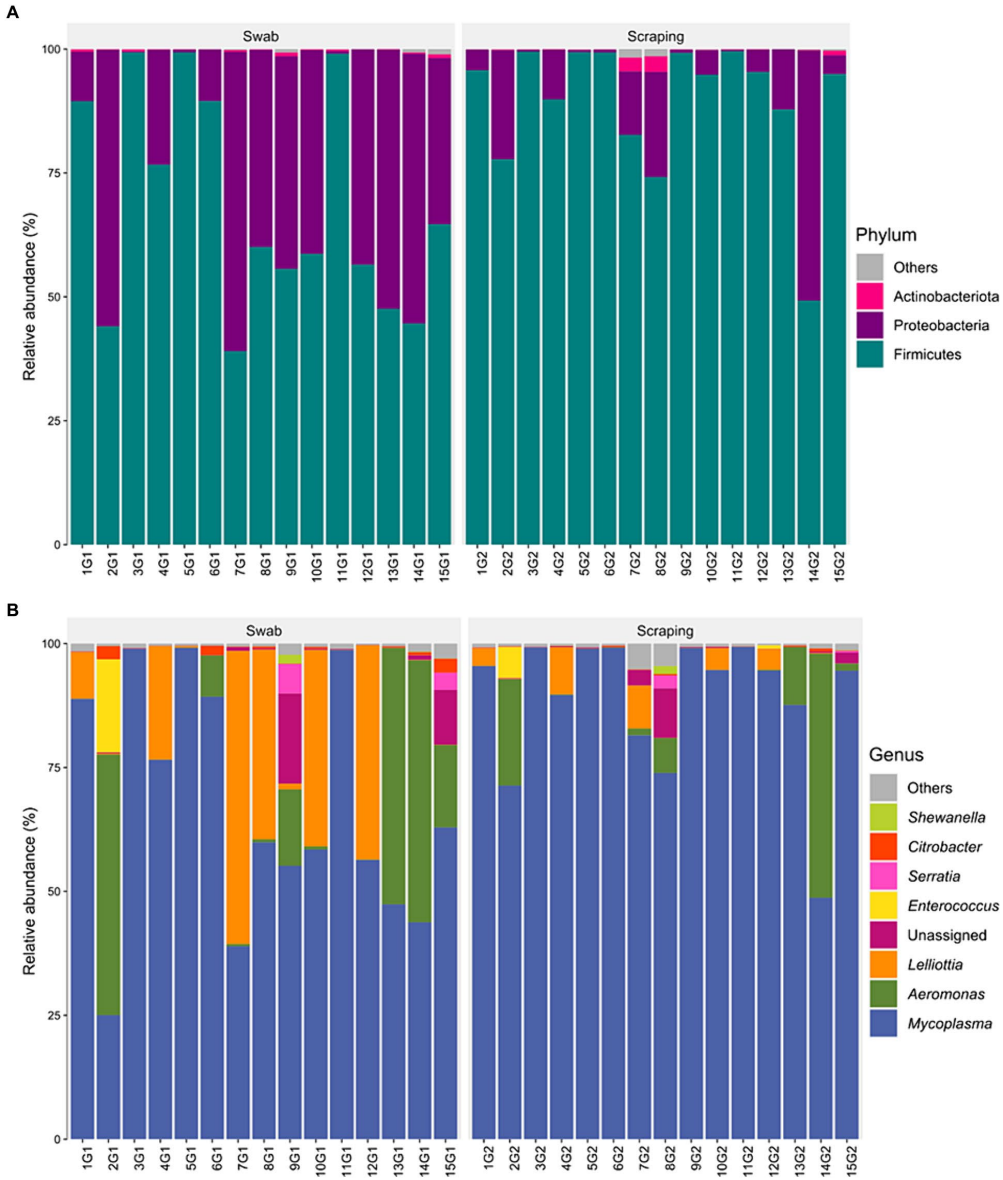
Relative abundance of the bacterial communities associated to the mucosa from the posterior intestine of rainbow trout (*Oncorhynchus mykiss*) collected by swabbing and scraping (*n* = 15 per sampling method), at the level of **(A)** phylum and **(B)** genus.

The bacterial communities recovered with both methodological procedures were also assessed, finding a clear dominance of the phylum Firmicutes (39–99.5% depending on the specimen considered; Figure 4A), mainly due to the high relative abundance of the genus *Mycoplasma* in all the samples (25–99.3%; Figure 4B). These results are in agreement with previous reports from rainbow trout (27, 70–72) and other salmonid species, such as Atlantic salmon (73), Chinook salmon (*Oncorhynchus tshawytscha*) (74), Arctic char (*Salvelinus alpinus*) (75), lake whitefish (*Coregonus clupeaformis*) (76), and whitefish (*Coregonus lavaretus pravdinellus*) (77), among others. The genus *Mycoplasma* can play different roles depending on the strain and on the host. Some strains belonging to this genus may act as pathogens in humans (78) and in a wide range of animals, most commonly in mammals and poultry (79). However, the high abundance of *Mycoplasma* is common when characterizing gut microbiota of salmonids and is not usually considered as an infection indicator when found in the intestine of fish (80). Indeed, many works have suggested a mutualistic relationship between *Mycoplasma* and salmonids, in which the bacteria belonging to this genus have health promoting effects for the host (72, 81). The high abundance of this genus on salmonids’ gut have been generally associated positively with an improved growth performance (73), carotenoid synthesis (82), biosynthesis of B vitamins and essential amino acids (83), disease resistance (84), and with an absence of pathogen infections and a healthy fish status (73), among others. Thus, many works have proposed *Mycoplasma* as a good biomarker to monitor the health status of salmonids in real-time using non-lethal sampling methodologies (73, 81). On the other hand, in a few studies, an increased relative abundance of *Mycoplasma* has also been correlated with opposite effects, such as an acute heat stress in rainbow trout (85) or with a parasitic infection in Atlantic salmon (86) or European whitefish (61). Therefore, it is important to remember the complexity of this genus when interpreting fish studies and, in this sense, the use of complementary characterization techniques that allow the identification of bacteria at lower taxonomic levels than the genus could shed light on the role of the different strains of this genus on salmonid guts in future studies.

**Figure 5:**
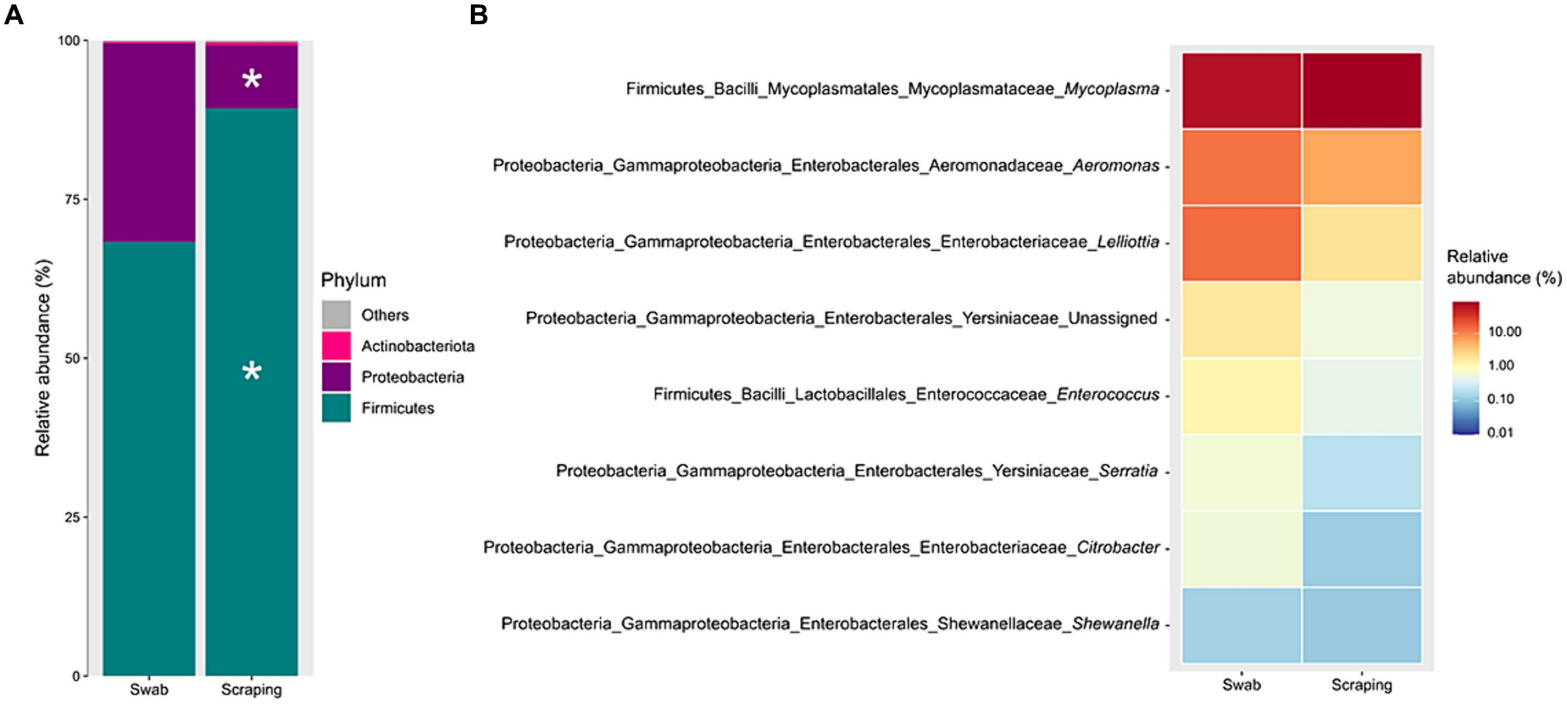
Significant differences (*p* < 0.05), marked by an asterisk, in the relative abundances of the bacterial communities associated to the mucosa from the posterior intestine of rainbow trout (*Oncorhynchus mykiss*) collected by swabbing and scraping (*n* = 15 per sampling method), at the level of **(A)** phylum and **(B)** genus.

When statistically comparing the taxonomic composition of the fish posterior intestinal samples between both procedures, a significant increase in the relative abundance of the phylum Firmicutes (*p* = 0.003), and a decrease of Proteobacteria (*p* = 0.003) were found when applying the scraping procedure with respect to the swabbing (Figure 5A). The increase in Firmicutes was directly attributed to the numerical higher relative abundance of the genus *Mycoplasma* (*p* = 0.051; Figure 5B). Furthermore, the decrease in the relative abundance of Proteobacteria was probably due to the cumulative effect of the numerical but not significant decrease of many genera belonging to this phylum like *Aeromonas*, *Lelliottia*, unassigned Yersiniaceae, *Serratia*, *Citrobacter*, *Shewanella*, and other genera with lower abundances (*p* > 0.05). Besides, the lower dominance of *Mycoplasma* on the swabbed mucosa was also associated with a higher inter-sample variability (Figure 4), which is in agreement with the dispersion observed among individuals for Bray-Curtis and weighted UniFrac distances.

A plausible reason behind the differences in phylum abundances and quantitative beta diversity metrics is that, considering the thickness of the tip used, the swabbing may recover only the bacterial communities present in the surface or mid parts of villi, while the scraping can also access to the basal region of the intestinal villi. A similar hypothesis was established in the work of Clinton et al. (57) to explain the differences observed when targeting the gills’ microbial communities of Atlantic salmon by swabbing or biopsy excision. The former authors proposed that the use of a tissue biopsy may reflect the microbiota from more ‘cryptic’ locations of the gills, which was expected to be more anaerobic, but even though some anaerobic genera were identified, the biopsy samples were highly dominated by one class: *Betaproteobacteria* (>80%), which are normally either aerobic or facultative anaerobic (87). Such results are certainly consistent with the present findings and, in particular, with the higher dominance of the genus *Mycoplasma* in the swabbed samples (*p* = 0.051), which are aerobic or facultative anaerobic microorganisms (88). A complementary and more likely hypothesis to explain the changes in abundance of this genus among procedures may be the differential pressure exerted by swabbing and scraping by the operator involved in sample acquisition. In this sense, *Mycoplasma* is a widespread genus in nature, which is usually found in two forms: as extracellular (or membrane-associated) bacteria, and as intracellular residents in the host cells (89). Consequently, this indicates that probably only the bacteria from the mucus layer and enterocytes’ apical surface were taken with the gentle movements applied with the swab, whereas the higher pressure exerted by the spatula when scraping the intestinal mucosa may recover the bacteria from the mucus, but also broke the epithelial cells and recover intracellular *Mycoplasma* (61).

In summary, both sampling procedures compared in the current study for the collection of mucosa-adhered bacteria in the posterior intestine of rainbow trout allowed a similar qualitative assessment of the species and phylogenetic features among samples. However, taxa abundances and beta diversity quantitative metrics showed differences, probably due to the targeting of more superficial communities with the swabbing procedure, and of cryptic and epithelial cell-dwelling communities with the scraping method. In this sense, swabbing allows a higher representation of low-abundance bacteria, which also play an important role in the host physiology as part of the autochthonous microbiota (66). Nevertheless, differences found between both methods may also be influenced by the fact that the same specimen was sampled twice, first with the swab, which might have changed the initial bacterial community, and then by scraping. The extent to which the differences were due to the repeated sampling or to the methodology applied itself could not be determined, even though results confirmed that both types of samples only differed in quantitative metrics, which seem to be a factor more dependent on the procedure applied rather than on the repeated collection of microbial content (57). With the information provided in this study, authors should decide which methodology to use in future works when defining their objectives. For instance, for comparative purposes with existent works or for meta-analysis studies, the sampling method applied in each work should be considered and respected as much as possible. Very few studies have aimed to characterize the fish autochthonous microbiota by non-lethal swabbing, and only using culture dependent-conventional identification techniques (90, 91). However, coupled with the progress of mass sequencing technology, this sampling procedure could be implemented as a non-lethal strategy to favor an aquaculture and wildlife research field closer to the 3Rs principle (92), avoiding more invasive or harmful procedures, such as overdose euthanasia. Beyond animal welfare, the application of swabbing may offer a long list of new experimental opportunities. Some of them could be the study of the effect of a stimulus (i.e., dietary change, induced stress, bacterial/viral challenge) on the fish microbiome under culture-controlled conditions, and the posterior correlation of the microbial communities’ composition over time within the same specimen with its survival, behavior, growth, or reproductive cycle in time-series experiment. With the existent development of different systems for individual fish identification (i.e., PIG tags, visible implant alpha tags, elastomers) (93), whether the application of non-lethal sampling procedures for microbiome assessment becomes a reality, as has happened in higher vertebrates, only depends on the researcher. Similarly, some of the above-mentioned opportunities that the swab sampling may offer could also be applied in the wild, such as the study of the correlation of the hindgut microbial dynamics and fish behavior over time, through telemetry and mark-recapture methods (69). Undoubtedly, the incorporation of this experimental approach into field studies would also open a new horizon of knowledge, ranging from microbial research in endangered species, or under legal protection and conservation concerns, to the study of the autochthonous microbiome of captive-bred species for future reintroduction and conservation in the wild.

## 4 Conclusion

This study demonstrates that the methodology employed to collect the mucosa-associated microbial communities from the fish gut can have an impact on the results obtained from 16S rRNA gene sequencing. While there were no differences in alpha diversity indices, and qualitative beta diversity metrics, significant differences were found in beta diversity quantitative assessment, and in phylum relative abundances between the conventional scraping method and the non-lethal swabbing method. Such differences may be the consequence of the lower accessibility of the flocked swabs to some bacterial communities present in the basal region of the intestinal villi and/or inhabiting the epithelial cells. Nonetheless, a higher representation of low abundant taxa was observed when applying swabbing rather than scraping. On the other hand, a higher variability for these quantitative measures was observed among individuals, despite not being a problem to discern significant differences between experimental groups in previous studies. Consequently, while scraping may be recommended for a more accurate microbial assessment of all intestinal areas, for example for diagnostic purposes, swabbing may allow a faster, less invasive, and non-lethal assessment of the fish posterior intestine with a multitude of potential applications in both aquaculture and wildlife studies, especially regarding intra-individual microbial tracking over time. The next step in implementing the use of this non-lethal strategy should focus on studying the optimal number of individuals required to evaluate significant differences between experimental groups when applying the swabbing procedure, the recovery time for microbial communities after this procedure in time-series experiments, which part of intestine could be characterized by this approach (only posterior or middle as well), and how long the nylon swab should be regarding different fish lengths and differing intestinal morphology of distinct species.

## Data availability statement

The datasets presented in this study can be found in online repositories. The names of the repository/repositories and accession number(s) can be found at: https://www.ncbi.nlm.nih.gov/, PRJNA1064675.

## Ethics statement

The animal study was approved by the Ethical Committee of Institute of Agrifood Research and Technology and the Generalitat of Catalunya (Spain). The study was conducted in accordance with the local legislation and institutional requirements.

## Author contributions

AR: Data curation, Formal analysis, Methodology, Software, Writing – original draft. ST: Investigation, Methodology, Writing – review & editing. EK: Validation, Writing – review & editing. KA: Investigation, Methodology, Writing – review & editing. MS: Validation, Writing – review & editing. EG: Conceptualization, Funding acquisition, Project administration, Supervision, Writing – review & editing.
