# Supplementary material for: Comparative study of the gut microbial communities collected by scraping and swabbing in a fish model: a comprehensive guide to promote non-lethal procedures for gut microbial studies
Source: Front Vet Sci. 2024 Mar 22;11:1374803. doi: 10.3389/fvets.2024.1374803 (PMC10997143; doi:10.3389/fvets.2024.1374803)

Supplementary Material

Supplementary Figure 1. Template rarefaction curves for observed ASVs from the mucosa of the posterior intestine of rainbow trout (*Oncorhynchus mykiss*) collected by swabbing and scraping (n = 15 per sampling method).

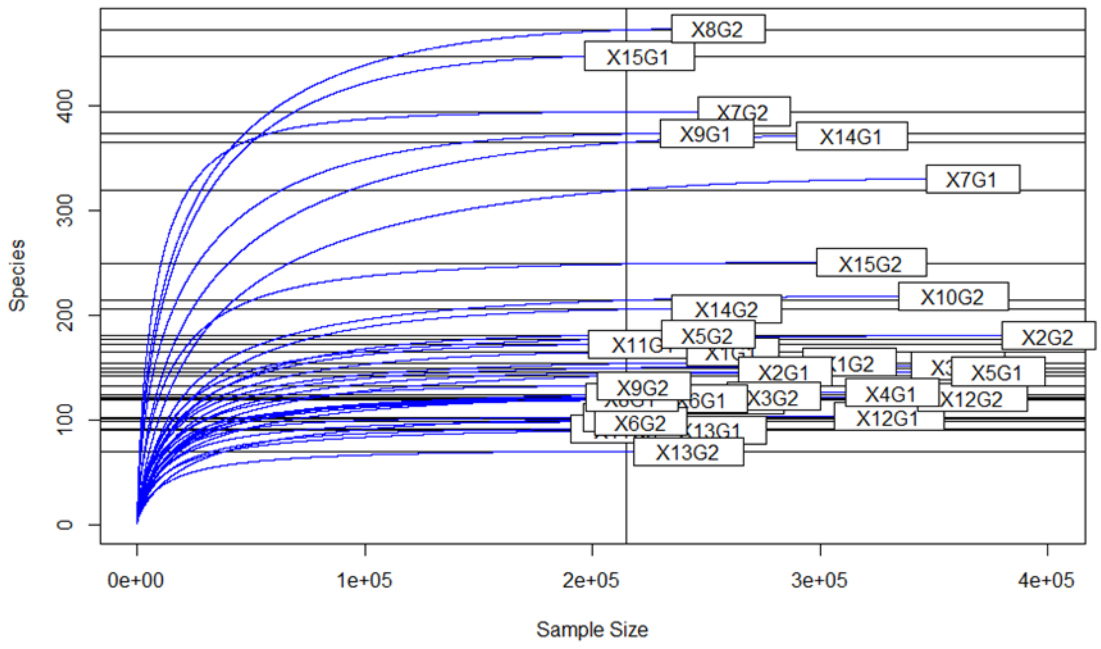

Supplement: Supplementary file 1 [file Data_Sheet_1.PDF]
